# Twins with Rheumatic Diseases

**Published:** 1969-10

**Authors:** A. St. J. Dixon


### TWINS WITH RHEUMATIC DISEASES

**A. St. J. Dixon**

It is almost 30 years since Professor Perry recorded observations on rheumatic heart disease in identical twins (Perry, 1940). Twins are nature's experiments, and a comparison of the concordance for a disease between monozygotic and dizygotic twins offers the possibility of assessing the contribution of inheritance to that disease. But the method has its difficulties, some relevant to all twin studies and some particular to chronic diseases. The "Bible" of gemellology is "Twins in History and Science" by Louis Gedda (1961), which covers all aspects including a review of twins in mythology and history. From Castor and Pollux to the Kray Brothers, twins have tended to attract more than their fair share of attention.

Twin pairs are of three sorts — (a) monozygous, like-sex (MZ), thought to have come from primary and equal subdivision of a single egg, and thus genetically (i.e. biochemically) identical, and commonly called "identical", although strictly they are not, (b) dizygous, like-sex (DZL) twins, commonly called fraternal twins, and (c) dizygous, unlike sex twins (DZU). Weinberg's formula (simplified) is:—

Number of MZ pairs=total number of twin pairs less 2 times number of DZU pairs.

The proportion of pairs which were MZ in a "Caucasian" population was 25 to 28% (Gates, 1952).

### *Are Twins representative?*

It cannot necessarily be assumed that twins are representative of the population in which they arise, or that MZ twins are identical one with another.

About two out of three pairs of MZ twins are monochorionic, that is, share a placental circulation. Imbalance of this shared circulation may lead to one foetus being starved, with considerable differences in birth weight (Benirschke 1961) and intelligence in later life (Kaelber and Pugh 1969). They may also possibly differ in things which go with intelligence (such as complaint threshold for pain and disability) or with birth weight (such as predisposition to other disease).

Even the assumption that MZ twins are biochemically identical has been challenged. The young of the nine-banded armadillo are monozygotic quadruplets, always of the same sex and always lying in the same position on the placenta. Yet, as Benirschke has shown, it is not possible for one of them to accept a skin graft from another. Monozygotic twins can even be of different apparent sex (Edwards *et al.,* 1966) when the ovum from a woman with a fertile sex-chromosomal abnormality is unequally shared at primary subdivisions.

The Registrar General's figures for pregnancies show that approximately one in eighty is a twin pregnancy and one in nine hundred a triplet pregnancy. About one in forty foetuses is thus a twin, but in the adult population we only find from one in sixty to one in eighty adults who has a living twin ("useful pairs"). The high antenatal and postnatal mortality of twins and the double risk of one of the pair dying is responsible. This means that a strong selection factor (not present in the control population) has to be considered in a twin study.

Twins as such are also more liable to Klinefelter's (Holub *et al.,* 1958) and Turner's syndromes (Hoefnagel & Benirschke, 1962) which may have associations with joint or bone disease. One of our twin pairs with a juvenile form of ankylosing spondylitis showed the Klinefelter characteristics.

Thus the first thing to do in a Twin Survey is to make sure that the twin rate for patients with the disease is the same as the twin rate in the population as a whole. This has been done in the study to be mentioned below; there is, in fact, no evidence that twins are more liable than non-twins to any of the common rheumatic diseases.

### TWINS AND FAMILIAL AGGREGATION OF RHEUMATIC DISEASES

All the main rheumatic diseases show an increased familial aggregation of cases. Aggregation is strongest for gout, haemophilia, and ochronosis, where the inheritance is strong. It is also strong in rheumatic fever, where streptococcal infection can obviously be familial. However, familial aggregation is also found in ankylosing spondylitis, Reiter's syndrome, the definable forms of osteoarthrosis (e.g. of the hip, or Heberden's nodes), and to a lesser extent in rheumatoid arthritis and psoriatic arthropathy.

In planning research on rheumatoid arthritis we need very much to know the contribution of inheritance to this increased familial aggregation. If we could say " There is no genetic component " we could eliminate straightaway half of the potential field for research. In adults the contribution of environment to disease can be studied by the method of spouses. Spouses share environment, social class, food, domestic infections, and so on. Over a large number of families a positive contribution by adult environment would show up as a greater incidence of husband/wife pairs affected than would be expected by chance. Childhood environmental factors can only be studied by the twin method. If rheumatoid arthritis (RA) were purely environmental, then MZ and DZ pairs would have the same frequency of RA. If RA were purely inherited then MZ pairs must both be affected if one is affected, but DZ pairs would have the same chance of both getting the disease as would a brother or sister. If RA were both environmental and inherited then the observed number of concordant cases would fall between those expected for a purely environmental disease and those for a purely inherited disease. However, if this were so, much larger numbers of pairs would be needed to assess the contribution of each factor.

A number of problems in case-finding can introduce bias or difficulty. The high mortality of MZ pairs in certain areas, for example Glasgow, is such that there is much more difficulty in finding " useful pairs ", especially MZ pairs. More serious is the problem of avoiding bias in case-finding. Simonds (1957) studied the apparent inheritance of tuberculosis; she found that tuberculosis appeared to be strongly inherited when twins were referred to her by those who knew of her interest. This was because MZ twins, especially if they go around together and get the same diseases, attract much more notice. But when *all* twins from a known population of patients were studied, the contribution of inheritance was shown to be minimal. Similarly, Court Brown and Doll (1961) state " examination of death entries relating to 51,425 children who had died of leukaemia or aplastic anaemia revealed only one example of the occurrence of one or other disease in both members of a pair of identical twins". But, of course, the doctor looking after that particular pair would take a lot of convincing that the double occurrence was only a chance coincidence. Moreover, the likelihood that such a concordant pair would get written up in the medical literature is very high, whereas discordant events in monozygous twin pairs normally never get published. The case reports of this type that have scientific value are not those which record a striking concordance of disease in a pair of twins, but the carefully observed pairs of which only one member is affected. Professor Perry's report is therefore still cited in the literature (and, incidentally, has been amply confirmed).

### PROBLEMS IN INTERPRETATION

Even when a twin study is mounted there are still problems. These include chimeraism, i.e. shared placental circulation in DZ twins, which interferes with subsequent blood grouping. We have already seen that MZ twins are not always identical, while DZ twins may appear identical simply by inheriting the same markers, e.g. the standard eight blood groups that are usually tested. The most important problem is that of delayed manifestation of the disease in the co-twin — especially so in chronic disease. How long does one wait before saying "The twin pair is discordant"? In one of our pairs, genetically identical by eight blood groups, hair and eye colour, dentition, finger print, and presence of congenital finger-clubbing, one patient was utterly rheumatoid and is now dead of the disease. The other has been followed over ten years and is absolutely normal. Another pair was concordant (i.e. both affected) at the time of the survey, but there had been an interval between the onsets of RA of about ten years. In yet another MZ pair, now 75, some twenty years have elapsed since one developed rheumatoid arthritis. The other has only osteoarthrosis. One of our DZU pairs was surveyed as discordant, but since the survey the previously unaffected sister has developed rheumatoid arthritis.

The other problem in interpretation concerns the different severity or different manifestation of disease in the co-twin. If the disease in a potential index twin is so severe that the patient dies, the normal co-twin will be missed in the case finding. If the manifestations of disease are different, is the disease concordant? Larsson and Leonhardt (1959) illustrate this problem. They report a pair of MZ twins of whom one had systemic lupus erythematosus but the other had hypergammaglobulinaemia only.

Since most of these problems are equally likely to affect MZ and DZ pairs we can in practice ignore them or get round them by judging concordance on separate manifestations rather than on total diagnosis.

### RESULTS OF A SURVEY

The Arthritis and Rheumatism Council Survey of Twins with Rheumatic Diseases was started in 1961 and is not yet published, although most of the data have been collected and the patients reviewed. Altogether 23 centres in this country and four in Holland co-operated. All patients attending each Centre were asked the question "Are you a twin?" If one said "Yes" the name and address of the co-twin, if alive, was recorded. All patients, twins and non-twins, had their diagnoses recorded so that we could work out the twin rates for any disease. X-ray survey, clinical examination of joints and sheep-cell tests were recorded in all twins. In like-sex twins, blood was sent for grouping. All " useful pairs " where both twins were alive and available, were identified and were revisited if possible. This was done irrespective of diagnosis.

By 1961 a total of 88 pairs of twins with rheumatoid arthritis had been discovered, and eventually we collected just under 200 pairs with rheumatoid arthritis. Previous to this study the total number of twins with rheumatoid arthritis in the world literature was less than ten. We can already say that the influence of inheritance on seropositive rheumatoid arthritis is not very great, although there must be some (see Table I). For osteoarthrosis, gout, spondylitis, rheumatic fever, etc., this method of case-finding did not collect enough individuals to lead to useful conclusions. These non-rheumatoid twins gave control data on the expected prevalence of positive sheep-cell tests. For seronegative rheumatoid arthritis there was no suggestion of inheritance.

Buchanan *et al.* (1967) studied five auto-antibodies, including a test for RA-factor in 105 healthy like-sex twin pairs. Analysis of the concordance rate in MZ and DZ pairs suggested that (in healthy people) the predisposition to form these five auto-antibodies was largely governed by environmental factors.

TABLE I

Incidence of Rheumatoid Arthritis in the Co-Twins of Index Twins with Seropositive Rheumatoid Arthritis

**Polyarthritis as found clinically**
**Grades 3-4**

| | | Observed | Expected | K (observed/expected) |
|---|---|---|---|---|
| Monozygous ......... | 15 pairs | 5 | 0.19 | 25 |
| Dizygous ........... Like sex | 39 pairs | 2 | 0.55 | 3.6 |
| Dizygous ........... Unlike sex | 69 pairs | 4 | 1.04 | 3.9 |

**Erosive Arthritis as found radiologically**
**Grades 3-4**

| | | Observed | Expected | K (observed/expected) |
|---|---|---|---|---|
| Monozygous ......... | 15 pairs | 4 | 0.12 | 33** |
| Dizygous ........... Like sex | 36 pairs | 2 | 0.30 | 6.7 |
| Dizygous ........... Unlike sex | 63 pairs | 4 | 0.61 | 6.6 |

**P. 0.01

Table I shows data taken from the interim findings of the Arthritis and Rheumatism Council Survey of Twins with Rheumatic Diseases. These concern 123 index twins with seropositive rheumatoid arthritis (R.A.) attending United Kingdom centres participating in the survey, by courtesy of Dr. J. R. Lawrence and the Council's Field Unit (Director: Dr. Philip Wood). Expected rates are assessed on some 3,000 controls. For all types of twins the number of concordant pairs observed was higher than expected, confirming increased familial aggregation. Only for grade 3-4 radiological arthritis did this achieve significance at the 1 in 20 level. K has a higher value for monozygous twins both for seropositive clinical rheumatoid arthritis and for radiological erosive arthritis, suggesting a weak inherited predisposition.

Therefore although we cannot absolutely rule out inherited (and therefore presumably genetic) factors in the aetiology of rheumatoid arthritis, these do not seem to be very important. It follows that the search for an inherited factor is not likely to be fruitful, but search for an environmental factor is. This is the importance of such a twin study. With hindsight, one can see how Perry's observation of discordance for rheumatic heart disease in MZ twins pointed clearly to a factor such as the haemolytic streptococcus, the importance of which was subsequently proven.

My thanks is due to Dr. J. S. Lawrence for help with case verification and analysis, and to the Arthritis & Rheumatism Council and its Field Unit, which supported this study.

**References**

Benirschke, K. (1961) N. York State J. Med. *61*, 1499.

Buchanan, W. W. (1967) Clin. & Exper. Immunol. *2*, 803.

Court Brown, W. M., & Doll, R. (1961) Brit. med. J. *1*, 981.

Edwards, J. H., Dent, T., Kahn, J. (1966) J. med. Genetics *3*, 117.

Gates, R. R. " Human Genetics " MacMillan, New York, 1952.

Gedda, L. " Twins in History & Science ", Oxford, Blackwell Scientific Publications 1961.

Hoefnagal, D., and Benirschke, K. (1962) Lancet *2*, 1282.

Holub, D. A., Grumbach, M. M., and Jailer, J. W. (1958) J. Clin. Endocrinol & Metabolism. *18*, 1359.

Kaelber, C. T. & Pugh, T. F. (1969) New Eng. J. Med. *280*, 1030.

Larsson, O. & Leonhardt, T. (1959) Act. Med. Scand. *165*, 371.

Perry, C. B. (1940) Arch. Dis. Child. *15*, 177.

Simonds, B. (1957) Eugenics Rev. *49*, 29.

